# Orthopaedic Resident Burnout: A Literature Review on Vulnerability, Risk Factors, Consequences and Management Strategies

**DOI:** 10.5704/MOJ.1907.003

**Published:** 2019-07

**Authors:** KP Wong, AK Kaliya-Perumal, JYL Oh

**Affiliations:** Department of Orthopaedic Surgery, Tan Tock Seng Hospital, Singapore

**Keywords:** burnout, depression, internship and residency, orthopaedics, surveys and questionnaires

## Abstract

**Introduction:** Orthopaedic surgery is physically demanding. Surgeons may have to work long unpredictable hours especially during residency training. This arduous task comes with the risk of burnout leading to negative repercussions to the surgeon and the patient. In view of strategising peer support, we intend to review the literature and analyse whether orthopaedic resident burnout is a global issue. We also intend to derive common strategies to tackle burnout at individual and organisational levels.

**Materials and Methods:** A literature search was carried out in the databases including PubMed, Scopus, SciELO, and Google Scholar to shortlist studies dealing with orthopaedic residency and related burnout. Those studies that used the Maslach Burnout Inventory (MBI) for quantifying burnout were collectively interpreted. Other studies were reviewed to analyse the vulnerability, risk factors, consequences and management strategies related to burnout.

**Results:** Among a total of 72 titles shortlisted, eight studies independently reported burnout among orthopaedic surgery residents/trainees and used MBI as a tool for assessing burnout. Based on the three subscales of MBI, 37.2% had high degree of emotional exhaustion (EE), 48% had high degree of depersonalisation (DP) and 33.1% perceived low personal accomplishment. This signifies the high prevalence of burnout among orthopaedic residents/trainees.

**Conclusion:** Burnout among orthopaedic surgery residents seems to be a universal problem. Risk factors could be multifactorial, influenced by clinical competency and work-home environment. This can be tackled at the individual level by being aware of burnout syndrome, involving in adequate physical activity and spending quality social time; and at the organisational level by duty hour limitation, professional appreciation and mentorship programme.

## Introduction

Burnout is characterised by loss of enthusiasm (emotional exhaustion), feeling of cynicism (depersonalisation) and reduced sense of personal accomplishment^1^. Surgeons, especially during early years of their career, work hard for long hours and make personal sacrifices to do so^2^. This arduous task can change life of individuals facing serious health problems^3^. However, this commitment comes with the risk of burnout leading to negative repercussions to the surgeon and the patient^2,4^.

In the recent years, there has been an increase in studies dealing with burnout among healthcare professionals, especially doctors^4^-6. More importantly, there is concern about burnout during the residency period which constitutes the early years of a budding career. Physical and mental wellbeing during this period is essential for cognitive functioning and developing decision-making abilities. The feeling of burnout at this stage can negatively impact patient care in many ways^7^-^10^. Therefore, identification of potential risk factors for burnout is necessary to tackle this issue; however, it could be multifactorial and may vary between specialities^11^.

For this literature review, we chose the orthopaedic speciality and studied the existing literature to collectively analyse studies dealing specifically with orthopaedic resident burnout. We concentrated on studies quantifying burnout using the Maslach Burnout Inventory (MBI) as it is considered the gold standard and deals specifically with the state of emotional exhaustion (EE), depersonalisation (DP) and personal accomplishment (PA) of the respondent^1^. We also reviewed studies using other well-being measures that have shown utility in various settings. Our intention was to gather information regarding who was affected, the risk factors, consequences and coping strategies. With key information from the available literature, we intend to provide common strategies that can be adopted by individuals and institutions to tackle burnout at the early stages.

## Materials and Methods

A research question was formulated as per the PICO (population; intervention; comparison; outcome) model. A list of words in English, Portuguese and Spanish were selected using DeCS (Descritores em Ciências da Saúde - Health Sciences Descriptors) and MeSH (Medical Subject Headings). A search strategy was constructed using logical operators.

The formulated search strategy was, ((orthopedics OR ortopedia) AND (“internship and residency” OR “Internato e Residência” OR “Internado y Residencia”) AND (burnout OR “Esgotamento Profissional” OR “Agotamiento Profesional” OR depression OR Depressão OR Depresión) AND (“Maslach burnout inventory” or “Surveys and Questionnaires” OR “Inquéritos e Questionários” OR “Encuestas y Cuestionarios”)).

This strategy was modified as appropriate to meet requirements of different databases such as PubMed, Scopus, SciELO, and Google Scholar. Studies dealing with orthopaedic residency and related burnout, published during the years 2000 to 2018, were shortlisted. For collective interpretation of results, only those studies using the Maslach Burnout Inventory (MBI) were selected. Data from other studies regarding resident burnout were also reviewed to analyse vulnerability, risk factors, consequences and management strategies related to burnout. Extracted data included author names, year, study design, sample size, sample characteristics, MBI score and proposed coping strategies if any.

## Results

Among a total of 72 titles shortlisted, only eight studies independently reported burnout among orthopaedic surgery residents/trainees, which used MBI as a tool for assessing burnout (Table I)^12^-^19^. These studies originated from different countries including Australia, France, Mexico, Netherlands and USA. Among the three subscales of MBI, emotional exhaustion (EE) and depersonalisation (DP) were considered by all included studies as direct reflectors of burnout; therefore, the rate of high EE or high DP was reported. However, rate of low personal accomplishment (PA) was only reported in five of the included studies. Collectively, among 973 orthopaedic residents/trainees, 37.2% had high degree of emotional exhaustion (EE) and 48% had high degree of depersonalisation (DP). Among 580 residents/trainees, 33.1% perceived low personal accomplishment. Prominent findings from other studies are discussed below.

**Table I:** Studies using MBI for assessing burnout among orthopaedic residents

Author (year)	Country	Sample size	High EE	MBI High DP	Low PA
Sargent MC *et al* (2009)	USA	384 residents	32 %	56 %	18 %
Arora M *et al* (2014)	Australia	51 trainees	45 %	35 %	43 %
van Vendeloo SN *et al* (2014)	Netherlands	105 trainees	16.2 %	11.4 %	-
Simons BS *et al* (2016)	USA	27 residents	29.6 %	37 %	25.9 %
Terrones-Rodriguez JF *et al* (2016)	Mexico	11 residents	54.5 %	72.7 %	45.5 %
Faivre G *et al* (2018)	France	107 residents	26 %	63 %	33 %
Hwang JS *et al* (2018)	USA	45 residents	75 %	83 %	-
Oladeji LO *et al* (2018)	USA	243 residents	18.9 %	25.6 %	-

MBI – Maslach Burnout Inventory; EE – Emotional exhaustion; DP – Depersonalisation; PA – Personal accomplishment

## Discussion

Burnout occurs invariably in all categories of healthcare workers^20^-^22^. Among doctors, it is reported across various specialities^23^-^25^. In our opinion, burnout follows a particular trend, that junior doctors report considerable burnout when compared to seniors. This was noted by Simons *et al* in a study among military orthopaedic residents where residents in their early years of training were reported to be at a higher risk of developing burnout^12^. Likewise, Sargent *et al*, reported that residents were considerably burnt out when compared to their faculty^14^. It is therefore understood that early career years is the time when the perception of burnout starts. If any preventive measures are thought of, it should be implemented at this stage.

Various studies had been done to identify risk factors for burnout. To highlight, Mendelsohn *et al* carried out a resident activity tracker evaluation (RATE) study, where the impact of work hours, sleep and physical activity on resident burnout was analysed^26^. This study inferred that surgical residents (general surgery and orthopaedics) worked more hours per week and had less sleep per day compared to other specialities. However, burnout rates were the same among all specialities. van Vendeloo *et al* reported evidence of burnout in a significant proportion of orthopaedic residents even though they followed an up-to-date curriculum with strict compliance to a 48-hour working week^19^. The studies did not provide conclusive of evidence of independent risk factors causing burnout.

However, independent risk factors were identified by Faivre *et al* in their multivariate analysis. Here, factors such as (a) medical errors within the past three months, (b) abnormal general health questionnaire (GHQ) 12 score and (c) living without a partner were found to independently correlate with occurrence of burnout^16^. Sargent *et al* studied risk factors in more detail by dealing with each subscale of the MBI^14,27^. Factors found to correlate with EE were anxiety about clinical competence, conflict between work and home, stressful relation with seniors and perception of work as stressful. Factors found to correlate with DP were increased work hours, stressful relation with nursing staff and increased anticipation of debt load at completion.

A high degree of burnout among doctors can lead to negative implications such as displaying hostile attitude toward patients, making medical errors and having difficult relationships with co-workers^7^-^9^. In addition to the deleterious effects on patient care, burnout also affects one’s own quality of life and subjective well-being^28^. This is a serious problem leading to job dissatisfaction, quitting intentions and even suicidal thoughts^29,30^. Burnt-out residents may not be able to perform duties to the best of their ability, which might add up to the stress of other colleagues who might eventually be affected due to burnout. Hence, it is important to prevent burnout from occurring rather than intervening when it has already occurred. This is for the betterment of both the individual and the organisation.

On reviewing the literature regarding management strategies, it is understood that there is no universal solution, mainly because the curriculum, work schedule and nature of work varies from place to place and between specialities. However, common strategies can be gathered from the available literature. We would like to divide this into personal strategies and professional strategies.

To start with personal strategies, Sargent *et al* stated that parenthood and increased satisfaction in talking to colleagues, friends and family has a positive impact on personal accomplishment^14,27^. This was also emphasised by Balch *et al* in their study. They identified key factors such as cultivating habits of personal renewal, emotional self-awareness and connection with colleagues that need to be encouraged^3^. In addition, physical activity also played an important role to reduce burnout^31^. To achieve both physical and social wellbeing, a good work- life balance is necessary^13^. Therefore, to avoid burnout, we encourage residents to give appropriate importance to social life, collegiality and physical fitness^32^.

Among the professional strategies studied so far, organisational support is considered to be the most important^33^. This includes (a) valuing one’s contributions and wellbeing, (b) providing adequate resources to facilitate performing their role and (c) assisting in overcoming crisis^34^. Providing such support reportedly decreased emotional exhaustion, thus leading to decreased burnout^35^.

To achieve all the above said, mentorship program is believed to play a significant role and is being implemented widely. Oladeji *et al* studied whether mentorship program as a part of residency training influenced burnout^18^. From their study, it was known that orthopaedic residents agreed that mentorship was a valuable part of training and needed to be encouraged. However, burnout was still reported probably due to mentorship quality and resident satisfaction^18^. Regarding the same, Sargent *et al* reported that mentoring can viewed to be of little help to reduce burnout^27^. Both these studies did not substantiate mentorship program as a definite tool to reduce burnout, however, it could be viewed as a valuable connection with a senior colleague, especially for counselling and encouragement at difficult times.

Other than this, Barrack *et al* found that resident duty hour limitation can have a positive impact on reducing burnout among orthopaedic surgery residents^36^. However, this is still controversial as various other studies describe that duty hour limitations did not influence burnout^26,37^. Only a longitudinal study on a larger group of residents can answer these questions regarding the best strategy to be adapted to overcome resident burnout.

## Conclusion

Burnout among orthopaedic surgery residents seems to be a universal problem. Evidence suggests that an average of 37.2%, 48% and 33.1% of orthopaedic residents suffer from high degree of emotional exhaustion (EE), high degree of depersonalisation and low personal accomplishment, respectively. Top risk factors for occurrence of burnout include poor clinical competency, mismatched work-home balance and lack of collegiality. This can be tackled at the individual level by being aware of burnout syndrome, involving in adequate physical activity and spending quality social time; and at the organisational level by duty hour limitation, professional appreciation and mentorship programme.
